# Little Evidence to Support the Risk–Disturbance Hypothesis as an Explanation for Responses to Anthropogenic Noise by Pygmy Marmosets (*Cebuella niveiventris*) at a Tourism site in the Peruvian Amazon

**DOI:** 10.1007/s10764-022-00297-9

**Published:** 2022-09-02

**Authors:** Emilie Hawkins, Sarah Papworth

**Affiliations:** Royal Holloway, University of London, Egham, Surrey TW20 0EX UK

**Keywords:** Ecotourism, Noise production, Tourist noise, Human–wildlife contact

## Abstract

The risk–disturbance hypothesis states that animals react to human stressors in the same way as they do to natural predators. Given increasing human–wildlife contact, understanding whether animals perceive anthropogenic sounds as a threat is important for assessing the long-term sustainability of wildlife tourism and proposing appropriate mitigation strategies. A study of pygmy marmoset (*Cebuella niveiventris*) responses to human speech found marmosets fled, decreased feeding and resting, and increased alert behaviors in response to human speech. Following this study, we investigated pygmy marmoset reactions to playbacks of different acoustic stimuli: controls (no playback, white noise and cicadas), anthropogenic noise (human speech and motorboats), and avian predators. For each playback condition, we recorded the behavior of a marmoset and looked at how the behaviors changed during and after the playback relative to behaviors before. We repeated this on ten different marmoset groups, playing each condition once to each group. The results did not replicate a previous study on the same species, at the same site, demonstrating the importance of replication in primate research, particularly when results are used to inform conservation policy. The results showed increased scanning during playbacks of the cicadas and predators compared with before the playback, and an increase in resting after playbacks of avian predators, but no evidence of behavior change in response to playbacks of human speech. There was no effect of ambient sound levels or distance between the playback source and focal animals on their behavior for all playback conditions. Although we find that noise can change the behavior of pygmy marmosets, we did not find evidence to support the risk–disturbance hypothesis.

**Supplementary Information:**

The online version contains supplementary material available at 10.1007/s10764-022-00297-9.

## Introduction

Although habitat destruction, hunting, and live capture are considered the primary threats to primate species (Chapman & Peres, 2001; Estrada *et al.,* 2017), other means of human contact with wildlife, for example through urbanization, agriculture, and infrastructure (Gordon, 2009; Hassell *et al.,* 2017; Krief *et al.,* 2017), cause other threats. For example, the development of ecotourism intentionally brings humans in contact with wildlife (Kight & Swaddle, 2011). Ecotourism is an increasingly popular industry which is an important income generator for many countries (Balmford *et al.,* 2009; Lindsey *et al.,* 2005; Terborgh *et al.,* 2002; World Travel and Tourism Council, 2007) and considered by many a valuable means to encouraging and funding conservation (e.g., Jacobson & Lopez, 1992). Studies of primate ecotourism have highlighted obvious deleterious effects, including habitat degradation and pollution (Wilson *et al.,* 2016) and the potential for human–primate disease transmission (McCarthy *et al.,* 2009; Palacios *et al.,* 2011), and more recently studies have focused on the impacts on primate behavior, specifically as a result of anthropogenic noise.

Anthropogenic noise can have deleterious effects on animals (Barber *et al.,* 2010; Kight & Swaddle, 2011; Shannon *et al.,* 2016); both physiologically, such as reduced cognitive outputs and increased cardiac stress (Anderson *et al.,* 2011; Chloupek *et al.,* 2009; Dooling & Popper, 2007; Owen *et al.,* 2004; Rabat, 2007) and behaviorally, such as reduced foraging and mating and increased vigilance (Blickley *et al.,* 2012; Fuller *et al.,* 2007; Payne *et al.,* 2015; Purser & Radford, 2011; Slabbekoorn & den Boer-Visser, 2006). Tourists can generate noise through movement and speech, and abiotic noise (e.g., from transport) can also increase as human presence increases. All primate species studied increased their vigilance behavior when tourist noise increased at a tourism site in Borneo (Leasor & Macgregor, 2014). In the same study, the number of proboscis monkeys (*Nasalis larvatus*) visible was negatively correlated with numbers of tourists and noise produced (Leasor & Macgregor, 2014). Pygmy marmosets (referred to in the study as *Cebuella pygmaea*, but now classified as *Cebuella niveiventris* following Garbino *et al.,* 2019) increased alert behaviors and reduced feeding and resting durations in response to playbacks of human speech (Sheehan & Papworth, 2019). Additionally, groups often fled in response to the stimulus, with a higher proportion of groups fleeing when the playback was louder (Sheehan & Papworth, 2019). Other studies of the effects of anthropogenic noise on primates show increased aggressive behaviors with exposure to human noise. For example, spider monkeys (*Ateles geoffroyi ornatus*) and male golden mantled howler monkeys (*Alouatta palliata palliata*) reacted aggressively when boats passed, and their faecal testosterone levels increased (Vanlangendonck *et al.,* 2015). Tibetan macaques (*Macaca thibetana*) showed heightened aggression in response to tourist behaviors with an auditory component, such as railing slaps, hand noise, foot noise and mouth noise (McCarthy *et al.,* 2009). A similar positive relationship was found between decibel levels produced by tourists and macaque threat response frequency (Ruesto *et al.,* 2010). However, we do not know if these responses are specific to humans or whether such reactions might be observed to any loud noise.

One possible explanation for why anthropogenic noise provokes behavioral changes is that animals perceive humans as a threat (even when hunting does not occur) and alter their behavior accordingly. This is known as the risk–disturbance hypothesis (Frid & Dill, 2002), where animals exhibit anti-predator responses when exposed to an anthropogenic stimulus such as human presence or anthropogenic noise. This can lead to fleeing or hiding, aggression, or increased vigilance (Dyck & Baydack, 2004; Eckhardt, 2005). These reactions take time and energy away from critical activities, for example foraging (Best, 1982) and mating, reducing reproductive success (Kight & Swaddle, 2011) with potentially long-term effects on the success of the species if these behaviors are frequently disrupted (Kight & Swaddle, 2011). The risk–disturbance hypothesis predicts similar responses to non-lethal stimuli as to predator stimuli (Frid & Dill, 2002). Some authors argue that predator-specific defenses and responses displayed by many animals refute the risk–disturbance hypothesis (Ghalambor & Martin, 2000). However, most species do react to stimuli such as loud noises or unknown approaching objects as a threat, as the risk of not reacting outweighs the cost of reacting (Frid & Dill, 2002) (i.e., the risk of death) (Bouskila & Blumstein, 1992; Johnson *et al.,* 2013). Studies looking at primate responses to predator playbacks show increased fleeing, resting, self-directed behaviors, alert postures, alarm calling and vigilance behaviors, and decreases in foraging activities (Campos & Fedigan, 2014; Gil-da-Costa *et al.,* 2003; Karpanty & Wright, 2007; Ouattara *et al.,* 2009; van Schaik & van Noordwijk, 1989).

Understanding whether the risk–disturbance hypothesis explains primate responses to human noise could inform decisions about possible mitigation strategies for the effects of anthropogenic noise. For example, habituation is often regarded as favourable for tourism and research, as it allows people to get closer to the animals being observed or studied (Bejder *et al.,* 2009; Johns, 1996; Lloyd & Ajarova, 2005). However, if responses to anthropogenic noise are analogous to predator responses, increased exposure may not have a habituating effect; some animals which experience increased exposure to humans instead show sensitization, where tolerance decreases, and responses are heightened (Bejder *et al.,* 2009). In such cases, mitigations other than habituation may be more appropriate. For example, howler monkeys rarely react to boats with the engine off (Vanlangendonck *et al.,* 2015), suggesting that turning off the engine when approaching known wildlife sites could be a simple mitigation strategy. However, if mitigation strategies based on behavioral observations lead to unnecessary restrictions or inappropriate recommendations, the tourist experience may be needlessly reduced (Griffin *et al.,* 2007) or primates may be exposed to unnecessary risks and stresses.

Distributed in the upper Amazon Basin of Peru, Bolivia, Brazil, Colombia, and Ecuador (Soini, 1988), pygmy marmosets are the world’s smallest monkeys. They are habitat specialists that reside in river-edge forests (Soini, 1988) and are specialized for exudate feeding (Jackson, 2011). Their territories are usually restricted to an area centered around one or several feeding trees, and they also feed opportunistically on fruit and insects (Jackson, 2011; Soini, 1988; Yepez *et al.,* 2005). They reside in small groups with one breeding female, her mate and her offspring of both sexes (Soini, 1988). Recently split into two species (Garbino *et al.,* 2019), they are a species with high potential for contact with humans and boats as they usually inhabit riverbanks of seasonally flooded forest (Snowdon & de la Torre, 2002). The small home range of the species makes them easy to relocate, making them popular with tourism guides, and means that habituation is not necessary to repeatedly encounter a particular group.

This study focuses on *Cebuella niveiventris*, found south of the Rio Solimões and Río Napo. Behavioral changes in pygmy marmosets in response to human speech led to a recommendation that tourists should be quieter, or ideally silent, when viewing these primates (Sheehan & Papworth, 2019). We aim to establish whether other conditions elicit a similar reaction in pygmy marmosets. Specifically, we test the risk–disturbance hypothesis by comparing whether responses to human stimuli are analogous to those to predator playbacks. If responses to humans are analogous to responses to predators, then the same behaviors should change in the same direction. We predict increases in time spent out of view, resting, self-directed, alert, alarm calling and vigilance, and decreases in feeding during the period after a playback of a predator in comparison to behavior before the playback.

## Methods

### Study Site

We collected data between 6 March 2019 and 6 May 2019, in and near the Area de Conservacion Regional Comunal de Tamshiyacu-Tahuayo (ACRCTT) in the north-western Peruvian Amazon (4.293519°S, 73.236237°W). The ACRCTT is one of the largest protected areas in Peru, set up in 1988 by the Rainforest Conservation Fund (RCF). The study site and surrounding areas experiences flooding annually (Kvist & Nebel, 2001), with the highest water levels in March and April. During the study, the water level at the site varied by approximately 2 m.

We used two sites that are close to one another, both of which are owned by the ecotourism company Amazonia Expeditions. Tahuayo Lodge is just outside the ACRCTT reserve. The Amazon Research Centre (ARC) is within the ACRCTT, a further 18 km upriver. The ARC is available for longer-stay tourists, but Tahuayo Lodge has more tourists. All our study groups were located within 20 minutes by motorboat from one of the lodges, in the same area as the previous study (Sheehan & Papworth, 2019).

Amazonia Expeditions offers personalized wildlife tours, and each tourist group is provided with a personal guide and boat, so group sizes vary (a single visitor will only be with their guide). Each tour group visits a different area, and visitors are encouraged to remain quiet but there are no formal rules for visitors viewing animals, and humans can potentially make a lot of noise in the presence of animals.

## Experimental Procedure

### Experimental Stimuli

We generated 60 unique stimuli for playback, with ten versions of each condition. We used three control conditions: no playback, cicadas, and white noise. During all trials, the research team did not talk and tried to make as little noise as possible. No playback refers to ambient sound with no noise intentionally emitted by the research team. We selected cicadas to represent a non-threatening stimulus, as the insect emits a loud call but does not pose a threat (Snowdon & Hodun, 1981). Cicadas may be included in pygmy marmoset diets as they eat various insects (Jackson, 2011; Soini, 1988; Yepez *et al.,* 2005) so the playbacks could potentially elicit scanning behaviors for hunting; however, we could not find any sources that specifically refer to pygmy marmosets consuming cicadas. We used white noise generated using version 2.3.3 of Audacity® recording and editing software to determine if noise itself elicits a response in pygmy marmosets. White noise, defined as having equal energy at all frequencies (Blumstein *et al.,* 2017), has been used as a control in similar studies (e.g., Jayne *et al.,* 2015), and is likely to be unfamiliar to pygmy marmosets.

For the experimental conditions, we chose predator calls and two types of anthropogenic noise: human speech and motorboat engines. We chose raptor calls as a predator stimulus as they are known predators of marmosets (Snowdon & de la Torre, 2002; Snowdon & Hodun, 1981). We generated ten versions of raptor stimuli from the calls of the harpy eagle (*Harpia harpyja*, *n* = 2), slate-colored hawk (*Buteogallus schistaceus*, *n* = 3), ornate hawk-eagle (*Spizaetus ornatus*, *n* = 2) and the black hawk-eagle (*Spizaetus tyrannus*, *n* = 3), as all are found around the study sites, although predation rates may differ between species. For human speech, we recorded five human males and five human females talking for 2 minutes each with a Sennheiser ME-66 Short-Gun Microphone linked to a Marantz PMD661 Field Recorder, to create ten different human speech stimuli. Although multiple tourists may talk at once in a group, we used a single individual for comparison with Sheehan and Papworth (2019). Motorboats are a common means of transportation in the area, so we used them as an anthropogenic source that did not involve human speech. We made ten recordings of motor engines, at different stages of travel, for example the engine starting, running continuously and stopping, and played a different recording to each group of pygmy marmosets. We edited all recordings to 24 s and removed background noise using version 2.3.3 of Audacity® recording and editing software.

Experiments were 300 s: 120 s of silence prior to the stimulus, a 24 s playback stimulus, then 156 s of silence. During the first 120 s, we abandoned trials if the focal individual disappeared for more than 20 s. Ideally, the 120 s period before the playback would be longer but when we were locating the groups, we found that longer observation times significantly reduced the chances of a successful trial, as we were unable to follow the subject easily from the canoe.

Changes in volume between 30 and 78 dB affect the behavioral responses of pygmy marmosets to playbacks of human speech (Sheehan & Papworth, 2019); therefore, we played all conditions at a mean of 60–70 dB (equivalent to human speaking volume: Lane *et al.,* 1961) when measured at 1 m. Volume during the playbacks fluctuated between 55 dB and 90 dB due to natural modulations in call patterns, particularly with raptor calls. We measured the mean and maximum decibels for each recording using a decibel meter at 1 m.

### Ambient Noise

Signals can be disrupted by other physical and acoustic features, such as the distance to the signal source, physical barriers in the environment, and ambient noise (Pijanowski *et al.,* 2011). Where possible, we kept these variables similar for each group (i.e., same distance, proximity to other trees), but in practice this was difficult as pygmy marmosets and the environment are unpredictable, so we measured ambient sound for each trial and included it in the analysis (mean 55.05 ± SD4.91 dB, *n* = 120). We measured ambient noise using a decibel meter immediately before and after each trial and calculated the mean. For four groups, anthropogenic sounds (e.g., tool use, music) were audible almost constantly, and were included in the ambient measurement. We conducted trials if these background sounds were ongoing, but abandoned trials if these sounds started at the same time as a playback stimulus.

### Study Groups

We located three groups using maps from Sheehan and Papworth (2019), and researchers and guides at the lodges located 15 additional groups. As some groups were close to each other we relied on the guides’ knowledge to ensure they were different groups. We included ten of these 18 groups in our study. Of the remaining eight, one did not have specific feeding trees so were not easily located, and four could not be observed or accessed due to high foliage density. We included the final three groups at the start of the study, but did not include them in analysis as we could not conduct one or more conditions for them during the study.

Our study groups had varying levels of human exposure, but all had experienced some sort of exposure to anthropogenic stimuli (boats, music, speech) prior to our study. Four groups (one of which was frequently visited by tourists) were in or near a village so were in hearing range of music, tool use, speech, etc. Another group near the ARC was visited by tourists, but less frequently than those around Tahuayo Lodge. Although we were careful to ensure we left 48 h before revisiting a group (detailed in ‘Data collection’ below) there is no guarantee that the groups were not visited by or exposed to people viewing other animals in the vicinity during this interim rest period.

### Data Collection

We played each group 1 x no playback, 1 x cicada, 1 x white noise, 1 x human speech, 1 x motorboat playbacks and 1 x predator calls. We randomized the order of the conditions for each group to control for potential temporal effects. We conducted trials 06:00–18.00 h every day. To reduce disturbance caused by our arrival, we approached groups by canoe or motorboat and switched the motor off 100 m away from the location. We set up the equipment before approaching the group and placed the speaker at the front of the canoe, with the team (two people) towards the back of the canoe. If we sighted no individuals, we left and returned after 2 hours. If we sighted individuals but did not conduct a playback, we did not visit the group again for 6 hours. Once we found an appropriate place to view the feeding tree and saw pygmy marmosets, we left a 2-minute period to allow the pygmy marmosets to adjust to our presence, before selecting a focal subject. We did not video record or note behaviors during the 2 minutes, and if the subject did not ignore our presence after this time (i.e., if they displayed researcher-directed vigilance or aggression) we aborted the trial.

The focal subject was the first marmoset we sighted when we arrived. All focal subjects were adults, but groups were not habituated, to minimize behavioral changes caused by increased exposure to humans, and we could not reliably identify the sex. There was no guarantee that we studied the same subject for each trial. We used a MiPro MA-303SB speaker and an Apple iPod Touch to conduct the playbacks, and recorded the focal subject using a Nikon D5200 SLR with a 55–300 mm lens. If the subject disappeared after the playback had started, we continued the trial, and recorded the subject as ‘absent’. We noted the time of the subject’s return if it returned within 20 minutes of the end of the recording. We could not ensure that we were observing the same subject after it disappeared. The subject was in view for the entire experiment in only five trials, but before playbacks commenced most movements out of view were for a short time, and subjects were in view for the whole playback in 37 experiments.

After the trial, we measured the distance from the speaker to the position of the marmoset at the start of the video, using a laser range finder. The mean distance was 9.31m ± SD2.31 m (range 5.36–14.53 m). After the completion of a successful trial, we did not attempt another trial with the group for at least 48 h. The mean return time to a group to present a new condition was 5 days (see supplementary material for more information).

## Data Analysis

We analysed all videos using Behavioral Observation Research Interactive Software (BORIS) (Friard *et al.,* 2016). We developed an ethogram (see supplementary material) and recorded the duration or occurrence of each behavior. We used nine behavioral categories: ‘Feeding’, ‘Resting’, ‘Social’ (grooming, play, aggression, and calling), ‘Self-grooming’, ‘Self-scratching’ ‘Alert posture’ ‘Researcher-directed aggression’ ‘Vigilance’ and ‘Movement’. ‘Vigilance’ is a difficult behavior to categorize, and we included three types of behaviors in this category: ‘general scanning behavior’, when an individual changed head/eye direction, ‘playback directed vigilance’ when the individual looked towards the playback source/researchers, and ‘head turns’, when the individual changed the direction of its head (Allan & Hill, 2018), as previous research found head turns, rather than scan duration, vary in response to predator models (Jones *et al.,* 2007). We combined general scanning behavior and playback-directed vigilance into ‘Total vigilance scanning behavior’. We split movement into ascent, descent, towards research team, away from research team, and behind tree. Of these, all apart from ‘behind tree’ involved the individual moving more than two body lengths without stopping for > 1 s in any direction. We included ‘Out of view’ (obscured, unknown location, and absent) and ‘Technical error’ in the ethogram (supplementary Table 1). We recorded the duration of all state behaviors, but ‘head turns’ and ‘calling’, classed as events, were recorded as counts not durations.

As the three time periods in the experiment (before, during, and after the stimuli) were different lengths and individuals were sometimes out of view, we standardized observed behavioral durations to seconds per minute of visible time, e.g., if an individual was visible for 114 s before the stimuli and was resting for 11 s, they were recorded as resting for 6 s per minute of visible time before the stimuli ($6=60\left(\frac{11}{114}\right)$). Event behaviors were standardized to rates (counts per minute). We assumed that behavior when individuals were in view was representative of behaviors when individuals were not in view. We calculated changes in behaviors between periods from these standardized durations. To determine if behavioral changes were immediate responses to the stimulus while it was playing or longer-lasting behavioral changes after the playback stopped, we compared the standardized behavior durations, and rates for during and after each playback stimulus to the behaviors recorded before the playback.

## Statistical Analysis

We conducted all statistical analyses using R version 3.6.3 (R Core Development Team, 2015) in R Studio Version 1.1.463. We tested for differences between conditions in behavior durations comparing before the playback to duration during and after the playback using generalized linear mixed effects models in R package lme4 (Bates *et al.,* 2015). We used mixed-effects models to test the effect of experimental condition (fixed effect), on duration of eight behaviors. For all behaviors except absence, we ran separate models to assess how the behaviors changed between before the playback and during and after the playback. To control for repeated testing of the same marmoset groups, we included group as a random variable. We included distance from the speaker and ambient sound as fixed effects to control for differences in the audible volume of the playbacks for focal subjects. We scaled both variables (mean = 0, SD = 1) so that estimates for the six conditions were generated at the mean distance from the speaker (9.3 ± SD2.9 m) at mean ambient sound (55.1 ± SD6.8 dB). We used binomial generalised linear mixed-effects models to test whether the subject moved, and whether they disappeared for more than 20 s after the playback started without returning during the trial. We used linear mixed-effects models to test for changes in scanning behaviors (including head turns), resting, and feeding. We ensured our dataset did not violate model assumptions using the package ‘Performance’ (Lüdecke *et al.,* 2020). We calculated conditional R^2^ (fixed effects) and marginal R^2^ (random and fixed effects) using the package ‘Performance’ (Lüdecke *et al.,* 2020). Some models had a singular fit. Further investigation showed this was due to a lack of variance between groups, resulting in random effects variance being calculated as zero. Removing the random effect from the model would have no effect on the estimated parameters (Pasch *et al.,* 2013), so we retained group as a random effect for comparability between analyses and to reflect the study design of repeated trials with ten groups. We calculated estimates of change in behavior in response to each condition from the models using the emmeans function in the ‘emmeans’ package (Lenth, 2021). Following the recommendations of Fidler *et al.* (2018) and Nakagawa and Cuthill (2007) we report and interpret both the *p* values and 95% confidence intervals.

## Ethical Note

The research was conducted with the permission of Amazonia Expeditions Research Center, Peru, meaning no permit was required. The study design was approved by the Royal Holloway Research Ethics Committee after being reviewed by the College Animal and Welfare Officer and United Kingdom Home Office Inspector assigned to Royal Holloway, University of London. The protocol included leaving 48 h between trials to reduce stress on the individuals studied. The authors declare that they have no conflict of interest.

### Data Availability

The datasets used during and/or analysed during the current study are available from the corresponding author on reasonable request.

## Results

### Overview

We attempted 81 trials over 13 groups and completed 60 trials successfully on ten groups. Three trials were unsuccessful because the focal subject moved out of sight for more than 20 s during the initial 120 s. We also abandoned trials before the playback if other people were present (*n* = 2), other animals were present and the marmosets moved out of sight (N = 6), or the equipment malfunctioned (*n* = 1). The following results are based on 60 trials from ten groups where we successfully applied all six conditions randomly.

**Fig. 1:**
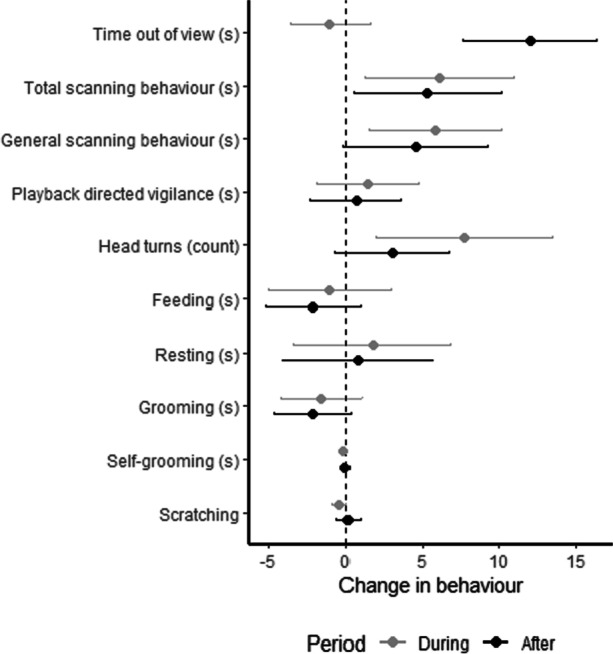
Mean and 95% confidence interval of changes in pygmy marmoset behavior during and after a playback stimulus compared to before the stimulus. Data are seconds per minute of visible time for all behaviors, except for head turns which are the rate per minute. Data are across all conditions (no playback, cicadas, white noise, human speech, motorboat, avian predators). This study was conducted in and close to the Area de Conservacion Regional Comunal de Tamshiyacu-Tahuayo, Peru, 2019.

Pygmy marmosets only called on four occasions during the 60 trials (once before the playback, once during, and twice afterwards). Due to the low occurrence, we grouped all calls into one category. We observed alert postures and researcher/playback-directed aggression once each in the 60 trials, and we excluded these behaviors from analyses due to their low occurrence. We observed scratching and grooming in very few trials (Fig. 1) and did not conduct analyses on these behaviors because the change in behavior was often zero due to low occurrence Table I.

**Table I:** Mean Duration and Standard Deviation of Pygmy Marmoset Behavior Before the Stimulus. The number of Trials Each Behavior was Observed in is Included. This Study was Conducted in and Close to the Area de Conservacion Regional Comunal de Tamshiyacu-Tahuayo, Peru, 2019

Behavior (seconds/minute of visible time except for heads turns, which is a rate)	Before	Number of trials behavior observed
Out of view	5.78 ± 6.44	55
Total scanning behavior	35.49 ± 16.91	60
General scanning behavior	26.66 ± 15.41	60
Playback directed vigilance	8.93 ± 10.51	52
Resting	21.84 ± 20.86	48
Number of head turns	26.45 ± 14.46	60
Feeding	10.74 ± 13.75	41
Grooming	3.64 ± 12.62	8
Self-grooming	0.12 ± 0.69	3
Scratching	0.75 ± 1.55	25

Data are seconds per minute of visible time for all behaviors except for head turns which are the rate per minute. Data are across all conditions (no playback, cicadas, white noise, human speech, motorboat, avian predators)

### Out of View and Absence

**Fig. 2:**
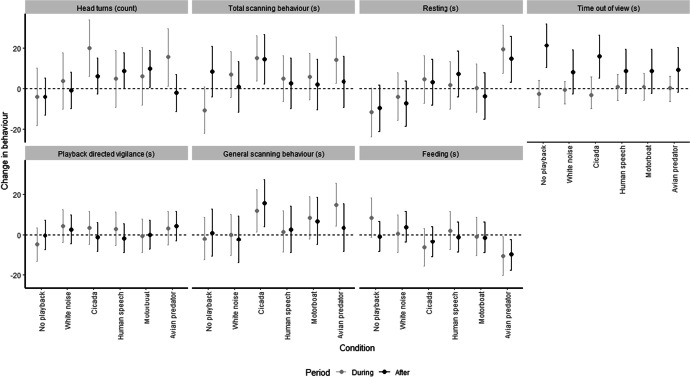
Mean and 95% confidence interval of changes in pygmy marmoset behavior during and after a playback stimulus compared to before the stimulus. Data are seconds per minute of visible time for all behaviors except for head turns, which are the rate per minute. Data are across all conditions (no playback, cicadas, white noise, human speech, motorboat, avian predators). This study was conducted in and close to the Area de Conservacion Regional Comunal de Tamshiyacu-Tahuayo, Peru, 2019.

**Fig. 3:**
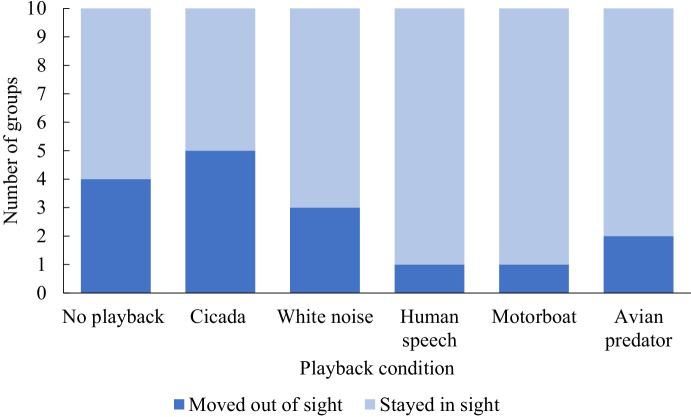
Number of pygmy marmoset groups where the subject was absent for more than 20 s after we started a playback and remained out of sight until the end of the trial. We conducted an equal number of trials on each group. The study was carried out in and close to the Area de Conservacion Regional Comunal de Tamshiyacu-Tahuayo, Peru, 2019.

Time spent out of view increased after playbacks relative to time spent out of view before (Fig. 1); there was not a significant difference between the playback conditions (Table II), although model estimates suggested that time out of view increased after the playback for the no playback and cicada conditions (Fig. 2). The cicada condition was associated with the highest number of subjects leaving the study area and not returning (*n* = 5), followed by the no playback condition (*n* = 4), and white noise (*n* = 3) (Fig. 3), but we found no significant differences between conditions (Table II).

**Table II:** Overall Results for Fixed Effects in Mixed Effect Models to Assess Behavioral Changes in Pygmy Marmosets in Response to Playback Experiments Relative to Behaviors Seen Before Playbacks. Playback Conditions are no Playback, Cicada Sounds, White Noise, Human Speech, Motorboat, and Avian Predators. This Study was Carried Out in 2019, Near the Area de Conservacion Regional Comunal de Tamshiyacu-Tahuayo, Peru

Behavior	Period	Explanatory variables	Test statistic	df	*P*	Marginal R^2^ (Conditional R^2^)
Out of view	during playback	condition sound distance	0.24 0.45 0.06	5 1 1	0.94 0.50 0.81	0.03 (0.10)
	after playback	condition sound distance	1.09 0.38 0.13	5 1 1	0.38 0.54 0.72	0.10 (0.18)
Absence	after start of playback	condition sound distance	5.94 0.06 0.22	5 1 1	0.31 0.81 0.64	0.18 (0.19)
Total scanning behavior	during playback	condition sound distance	**2.78** 0.18 3.31	**5** 1 1	**0.02** 0.67 0.07	0.20 (0.24)
	after playback	condition sound distance	0.81 0.11 0.05	5 1 1	0.55 0.74 0.82	0.06 (0.16)
Playback directed vigilance	during playback	condition sound distance	0.72 0.33 0.10	5 1 1	0.61 0.57 0.75	0.07 (0.07)*
	after playback	condition sound distance	0.41 3.33 3.00	5 1 1	0.84 0.07 0.09	0.11 (0.11)*
General scanning behavior	during playback	condition sound distance	1.87 2.02 2.09	5 1 1	0.12 0.16 0.15	0.16 (0.24)
	after playback	condition sound distance	1.31 1.50 0.20	5 1 1	0.28 0.23 0.65	0.11 (0.22)
Head turns	during playback	condition sound distance	1.60 0.68 0.62	5 1 1	0.18 0.42 0.44	0.13 (0.16)
	after playback	condition sound distance	1.79 0.45 0.50	5 1 1	0.13 0.50 0.86	0.13 (0.13)*
Resting	during playback	condition sound distance	**2.97** 0.03 0.90	**5** 1 1	**0.02** 0.85 0.35	0.20 (0.20)*
	after playback	condition sound distance	**2.76** 1.39 0.24	**5** 1 1	**0.03** 0.24 0.93	0.21 (0.21)*
Feeding	during playback	condition sound distance	1.94 0.13 2.56	5 1 1	0.11 0.72 0.12	0.15 (0.17)
	after playback	condition sound distance	1.40 0.11 0.01	5 1 1	0.24 0.74 0.91	0.11 (0.11)*

Significant results at alpha = 0.05 are shown in bold. We derived test statistics (F for all models except absence, where the test statistic was χ^2^), df and *p* values using ‘Anova’, and R^2^ values using the ‘R2’ function in the package ‘performance’. R^2^ values with asterisks indicate models where differences between groups explained no variance (see methods for details)

### Vigilance Responses

Across all trials, total scanning behavior increased during and after the playback compared to before it (Fig. 1). Total scanning behavior was significantly different between the playback conditions during the playback but not after it (Table II). The cicada and predator conditions were associated with an increase in total scanning behavior during the playback, but only the cicada condition was associated with an increase in total scanning after the playback (Fig. 2). Across all trials, general scanning behavior increased during the playback, but we found no consistent changes in playback directed vigilance (Fig. 1). Overall, the results of the mixed effects model did not reject the null hypothesis of no difference in general scanning behavior between conditions (Table II), but the 95% confidence intervals suggest an increase in general scanning behavior during the playback for predator and cicada conditions and an increase in cicada condition after the playback (Fig. 2). We found no evidence for differences in playback directed vigilance between conditions either during or after the payback (Table II and Fig. 2). Across all trials, the number of head turns increased during the playback compared to before it (Fig. 1). We could not reject the null hypothesis of no differences in head turns between conditions (Table II), but the estimates and 95% confidence intervals indicate an increase in head turns for the predator and cicada conditions during the playback and an increase in head turns after it in the motorboat condition (Fig. 2).

### Resting and Feeding

Across all trials, there was no consistent change in duration of resting or feeding during either period either during or after the playback (Fig. 1), but resting duration differed between conditions in both periods (Table II). Resting increased in both periods for the predator condition, but not for other conditions (Fig. 2). We could not reject the null hypothesis of no difference between conditions for either period (Table II), but the estimates and 95% confidence intervals indicate a decrease in feeding for the predator condition (but no other condition) in both periods (Fig. 2).

## Discussion

### Response to Playback Experiments

We found differences between conditions in changes in total scanning behavior duration during the playback relative to before the playback, and differences between conditions in resting behavior during and after the playback. Specifically, we observed increases in resting behavior only in response to avian predator stimuli, and increases in total scanning behavior in response to cicada and avian predator stimuli. Although mixed effects linear models did not reject the null hypothesis of no difference between conditions for any other measured behavior, model estimates and 95% confidence intervals suggest other possible changes in behavior. These are increases in time out of view in response to no playback and cicada playbacks, increases in head turns in response to cicada, motorboat and avian predator playbacks, increases in general scanning behavior in response to cicada and avian predator playbacks, and decreases in feeding behavior in response to avian predator playbacks. We found no effect of distance between the subject and the playback source, or ambient sound levels on behaviors between conditions for each group.

### Support for the Risk–Disturbance Hypothesis

Behavior change differed between the human speech and avian predator playback conditions. In the avian predator condition, total and general scanning behavior, head turns and resting increased, and feeding decreased, during the playback, while resting increased and feeding decreased after the playback. These behaviors are consistent with anti-predator behaviors reported for other primates (e.g., van Schaik & van Noordwijk, 1989). However, we observed no changes in behavior in the human speech condition. Thus, our findings do not support the risk disturbance hypothesis. Given that many primates have different responses to different types of predators (e.g., Ouattara *et al.,* 2009; Papworth *et al.,* 2008; Sheehan & Papworth, 2019), it is possible that pygmy marmoset responses to humans are analogous to their responses to non-avian predators that we did not test. However, we did not observe any behavioral changes in response to human speech, and it would be surprising if pygmy marmosets did not change any of their behaviors in response to a predator. These findings suggest that pygmy marmosets do not perceive humans as similar to other predators. Our findings accord with a study of chacma baboons (*Papio ursinus*), which perceived human observers as equivalent to social threats even when habituated (Allan *et al.,* 2020) but did not react as they would to a predator, suggesting that the risk disturbance hypothesis may not describe primate responses to humans, but more studies are needed across other primate species.

Overall, the greatest evidence for consistent behavioral changes was in response to the predator and cicada conditions. Changes in vigilance behaviors (scanning and head turns) in response to these conditions were identical during the playback. This could be due to our definitions of ‘vigilance’ behaviors, with our classifications of total and general scanning behavior potentially encompassing arousal and scanning behaviors. Pygmy marmosets are unlikely to view cicadas as a threat (and as pygmy marmosets eat insects, they may form part of their diet, Jackson, 2011), further suggesting that the observed behavioral changes do not constitute a generalised response to perceived risk. Instead, it may be that the behavioral responses we observed (increased scanning behaviors and head turns) indicate increased arousal, as focal individuals attempt to hunt cicadas.

We observed self-directed behaviors and calling in fewer than half of all trials. The low levels of these behaviors were unexpected: pygmy marmosets call in response to raptor stimuli (Snowdon & de la Torre, 2002; Snowdon & Hodun, 1981), and self-scratching and other self-directed behaviors are important indicator behaviors of anxiety in other primates (Castles *et al.,* 1999; Maréchal *et al.,* 2016); so we expected them to increase in response to disturbing stimuli. Furthermore, self-directed behaviors increase with tourist density in Tibetan macaques (Matheson *et al.,* 2006). We also predicted changes in social behaviors during the trials, as social behaviors such as grooming have an important role in anthropoid primate communication (Dunbar, 2010). It is possible that we missed some of these behaviors, for example if the individual was facing away from the camera (although calls should have been heard), but this was unavoidable as we could not always follow the individual from the canoe.

The lack of self-directed behaviors (scratching, grooming) and calls raises questions about whether the experimental setup realistically replicated the stimuli we intended. For example, sounds of an avian predator may only be perceived as threatening and elicit an alarm call if heard from above and accompanied by a sighting (although previous studies of primate responses to avian predators have elicited anti-predator calls to playback experiments at ground levels without models, e.g., Papworth *et al.,* 2008). The visible presence of the researcher may also have affected our results. For example, less habituated or tolerant (and more fearful) groups and individuals may have been harder to locate or may have fled before we could begin experiments. Moreover, we aborted trails when the subject displayed researcher-directed vigilance or aggression before the trial started. Therefore, subjects included in the study are probably more habituated or tolerant individuals or groups which are less likely to respond to anthropogenic disturbances, which is supported by the lack of response to human stimuli and the high levels of exposure to anthropogenic noise (at least for some groups). This tolerance of humans could also affect their response to predators, for example through the human-shield effect, where humans deter less tolerant predators so prey species can use humans as a shield against predation (Geffroy *et al.,* 2015).

To here The uncertainties caused by the presence of humans in the experimental setup could be overcome by repeating this experiment using the automated behavioral response system designed by Suraci *et al.* (2017). The system records behaviors using camera traps and has linked speakers which play audio stimuli when the camera trap is tripped. In addition to removing human presence, multiple autonomous systems would mean multiple groups could be studied concurrently, increasing capture of rarer behaviors. This approach could be used to repeat trials of the same condition multiple times for the same group. This would allow better understanding of variation between and within groups, which some models in this study failed to approximate (indicated by the models with a singular fit), and the use of more complex models which include other covariates such as differences between groups in exposure to anthropogenic disturbance. This increased sampling would also allow us to test multiple hypotheses which describe animal responses to humans (for example, contrasting the risk–disturbance hypothesis and the human-shield hypothesis), and allow finer investigation of some of the conditions, e.g., differences in responses to male and female human speech, or different avian predators. Larissa Barker planned to start this research in March 2020 but it was delayed due to COVID-19 travel restrictions.

A previous study at the same site as our study found decreases in visibility, resting, feeding, and increases in time holding an alert posture when pygmy marmosets were played recordings of human speech (Sheehan & Papworth, 2019). Our study did not replicate these findings. Instead, we found no significant changes in behavior in response to a playback of human speech, and only one instance of an alert posture. Another striking difference between the two studies is that individuals only moved out of sight in trials with human speech in Sheehan and Papworth (2019), but this occurred in all conditions in this study, including four out of ten silent (no playback) trials. Sheehan and Papworth (2019) interpreted their finding of no movement out of sight in the silent condition as a lack of response to human presence. If we apply the same interpretation in this study, it suggests the presence of the research team affected the results as some individuals moved away during the ‘no playback’ condition and time out of view increased in the period after the playback. Furthermore, we observed few behavioral changes in playbacks of human speech and motorboats, and only one group moved completely out of sight, the lowest incidence across all conditions, suggesting that these conditions were the least disturbing to the pygmy marmosets. Although not an exact replication of Sheehan and Papworth (2019), this study was a ‘conceptual replication’ (Nosek & Errington, 2020) and we anticipated that we would replicate the results of Sheehan and Papworth (2019). The differences in results may be due to differences in protocols. For example, Sheehan and Papworth (2019) had a 5-minute rest period and compared behavior before and after the playback start, whereas this study had a 2-minute rest period and separated behavior during and after the playback to better understand when behavior changed.

Temporal or spatial differences between the groups or individuals in the studies may also have led to the differences between the two studies. Pygmy marmoset tolerance of or sensitivity to human speech may have changed over the 2 years between the studies. Both studies were executed with ten pygmy marmoset groups, but only three groups were included in both studies. These groups probably differed in their exposure to anthropogenic and predator disturbances, which could affect their responses (Stankowich, 2008; Stankowich & Blumstein, 2005), as well as other covariates which could affect anti-predator behavior and are not included in the analyses, such as group size (e.g., Beauchamp, 2017). Likewise, each group was exposed to multiple conditions, but we studied a single individual from the group, and we do not know if this was the same individual each time (it is likely that we studied different individuals) and individual differences in responses could also impact the results. For example, males and females in other primates have different responses to predators (e.g., Ouattara *et al.,* 2009). The impact of individual variation could be explored through identification of individuals within groups and repeated presentation of conditions to the same individuals.

Establishing whether human speech is a source of disturbance for primates has practical implications for primate tourism. Sheehan and Papworth (2019, p. 1) suggest that ‘negative tourist impacts can be reduced by encouraging tourists to refrain from speaking in the presence of visited primate groups’, but our findings do not support theirs, although together these two studies could be interpreted as showing the possible range of behavioral responses to humans. Replication is receiving increasing attention in other disciplines (e.g., psychology, Open Science Collaboration, 2015), and our failure to replicate the results of a previous study on the same species, at the same site, demonstrates the importance of replication in primate research, particularly when results might be used to inform conservation policy.

Conservation is a ‘crisis discipline' (Soulé, 1985), where action is often taken in response to a negative impact, not as a preventative measure. Without knowing why there are differences between this study and the study by Sheehan and Papworth (2019), there is an argument for taking a precautionary approach and continuing to recommend that noise be minimized by primate tourism enterprises. However, mitigation strategies require resources to implement and monitor for effectiveness, which could be better used elsewhere. Monitoring the effectiveness of interventions is particularly important for primate conservation, as a recent synopsis of interventions found no evidence evaluating the effectiveness of 59% of conservation interventions suggested by an advisory board (Petrovan *et al.,* 2018). Following the recommendations of Sheehan and Papworth (2019), Hawkins (2019) evaluated whether informing tourists about behavioral changes by pygmy marmosets in response to human speech changed a tourist’s self-reported behavioral intentions. However, there was no difference in responses between the experimental and control groups, suggesting education may not be an effective tool to change tourist behavior in this context (Hawkins, 2019). Given the uncertainty about the impact of tourist noise on pygmy marmosets, and the lack of impact of this mitigation strategy on behavior change of tourists, the available evidence does not support further recommendations to reduce tourist noise at this site and this may be a poor use of resources. Furthermore, the effects of such an intervention on other animals is unknown and could have undesirable effects — for example, less tolerant species and individuals may use tourist noise as a prompt to temporarily move away from an area.

In terms of future research, one aim could be to establish why these differences in results occurred, to ensure that recommendations for primate tourism are based on sound evidence, and to better inform future studies of other species. Locating the groups from both Sheehan and Papworth (2019) and this study to conduct further experiments to investigate the role of tolerance, habituation, and group level differences in responses to anthropogenic noise would be one approach. Locating other groups to ensure a larger sample size, and repeated trials of each condition for each group, would improve our understanding of the impacts of human noise on pygmy marmosets and allow robust recommendations for primate tourism to be made. For example, if the reduction in response to human speech in this study compared to Sheehan and Papworth (2019) is due to temporal changes in responses because of habituation, this could lead to recommendations for a pattern of visits where fewer groups are habituated and visited more often, reducing disturbance at a population level. However, even groups of primates that are repeatedly visited show no signs of habituation to tourists (Treves & Brandon, 2005), and even if habituation is successful, the increased closeness of habituation comes with potential health issues such as human to primate disease spread (Woodford *et al.,* 2002), and a reduction in tourist awareness of potential threats to the visited animals (Stone & Yoshinaga, 2000).

### Conclusion

Our results did not support the risk–disturbance hypothesis, and we did not find that human speech changed pygmy marmoset behavior. Nevertheless, further studies of this nature are important, as human speech has previously been shown to have negative impacts on pygmy marmoset behavior (Sheehan & Papworth, 2019), and we only investigated immediate, rather than long-term, impacts of anthropogenic noise. Animals are often negatively affected by human interactions (Barber *et al.,* 2010; Kight & Swaddle, 2011; McCarthy *et al.,* 2009; Shannon *et al.,* 2016; Vanlangendonck *et al.,* 2015), so there is an argument for taking a precautionary approach and continuing to recommend that noise be minimized by primate tourism enterprises, but these changes should be monitored to provide evidence to support the value of this approach. Further studies on the impact of anthropogenic noise on primates and complementary studies on mitigation methods could play an important role in establishing a sustainable future for ecotourism.

## Supplementary Information

ESM 1 (DOCX 22 kb)
