# Supplementary material for: Little Evidence to Support the Risk–Disturbance Hypothesis as an Explanation for Responses to Anthropogenic Noise by Pygmy Marmosets (Cebuella niveiventris) at a Tourism site in the Peruvian Amazon
Source: Int J Primatol. 2022 Sep 2;43(6):1110–32. doi: 10.1007/s10764-022-00297-9 (PMC9438364; doi:10.1007/s10764-022-00297-9)
Supplement: Supplementary file 1 — (DOCX 22 kb) [file 10764_2022_297_MOESM1_ESM.docx]

**Supplementary Material**

# *Pygmy marmoset protocol*

## Preparation

The recordings were collected and recorded prior to fieldwork. Each were set at 60db-70dB measured at 1m on average, with no higher than 85dB for peaks of call modulation. Each stimulus was a recording with a 24 second sound (or silence) with 2 minutes silence before and 156 seconds of silence after. The marmoset groups were located and identified at the start of the fieldwork and each group were visited and studied in a cyclic pattern to leave sufficient time between each trial to reduce stress. The recordings were allocated randomly, but each group were played a specific type of recording once i.e. each were played 1x predator sound, 1x cicada sound, 1x white noise, 1x motorboat sound and 1x human speech recording.

## Trial protocol

The trial group was approached by canoe, if the motorboat was required to travel to the location the engine was switched off at least 100m from the group and then we transferred to a canoe, to reduce the effect of our presence. Once the location was reached (the feeding tree) the initial time of sighting was recorded. If no individual was recorded within 30 minutes of arrival the trial was abandoned, and the group was approached at the next opportunity. Two hours were left if no individuals were sighted but six hours were left between trials if individuals were sighted but the trial could not be completed.

The equipment was quietly set up before reaching the group and then a rest period of 2 minutes started once an individual was sighted. Once the rest period was over, the trial began if an individual was still in sight. The study used a handheld camera to record the trial, and the individual was recorded for 2 minutes prior to the sound playback, during the 2 minutes of silence included in each stimulus before the experimental condition, allowing uninterrupted recording without the need to play the stimulus after 2 minutes. The sound then automatically played for 24 seconds after the 2 minutes of silence. Once the stimulus had ended, the individual was continued to be recorded until the total recording time reached 300 seconds. The period of silence after the sound allowed the completion of the trial without the need to turn of the speaker after the stimulus had ended and prevented automatic skipping to the next track. Each trial focused on one individual, if it did not disappear from site for more than 20 seconds during the initial 2 minutes of recording.

The data recorded at the location included times and date, GPS (group ID), treatment, and starting distance of individual from speaker (measured after the trial). Movement before, during and after the trial was recorded along with the direction (movement was only considered as a response to a stimulus if the individual moved more than 2 body lengths without stopping for >1 second in any direction).

## Absent protocol

If during the initial 2 minutes, before the commencement of the trial, the focal individual disappeared from view and did not return within 20 seconds, the recording was stopped. The trial then waited for the individual to return or failing this selected another individual.

If the individual disappeared from view after the playback had started, the trial continued, and the individual was recorded as ‘absent’. The time was then recorded of the individuals return (if within 20 minutes following the end of the recording).

## Analysis protocol

The behavioral observations were analyzed from the recording in BORIS and categorized using the following ethogram. The observations were be separated into ‘before playback’ ‘during playback’ ‘after playback’. The duration of each behavior was also recorded in BORIS.

**Supplementary table 1**. Ethogram describing how each behavior was categorized during observations of pygmy marmosets in the Peruvian Amazon between March and May 2019.

| Behavior group | Behavior | Description |
| --- | --- | --- |
| Movement | Ascent | Individual moves upwards more than 2 body lengths without stopping for >1 second. State behavior. Measured in seconds. |
|  | Descent | Individual moves downwards more than 2 body lengths without stopping for >1 second. State behavior. Measured in seconds. |
|  | Movement away from speaker/researcher | Individual moves away from speaker or researcher by more than 2 body lengths without stopping for >1 second in any direction excluding ascent or descent. State behavior. Measured in seconds. |
|  | Movement toward speaker/researcher | Individual moves toward the speaker or researcher by more than 2 body lengths without stopping for >1 second in any direction excluding ascent or descent. State behavior. Measured in seconds. |
|  | Movement behind tree | Individual moves behind the tree once stimulus has started. State behavior. Measured in seconds. |
| Social | Grooming | The individual spends time grooming, or being groomed by, other members of the group. (identified when individual searches other’s fur using hands or mouth). State behavior. Measured in seconds. |
|  | Play | Individual involved in play behavior, includes chasing and hugging of other individuals. State behavior. Measured in seconds. |
|  | Aggression | Individual is involved in aggressive behaviors including chasing and biting, State behavior. Measured in seconds. |
|  | Calling | Any calls are identified my open mouth and noise emission. Point behavior. |
| Resting | Resting | The individual remains stationary for >1 second without any secondary behaviors (e.g. feeding / grooming, alert posture) aside from vigilance. State behavior. Measured in seconds. |
| Feeding | Feeding | Individual is foraging or feeding, identified by placing limbs to mouth or mouth to tree and by visibly masticating. State behavior. Measured in seconds. |
| Alert posture | Alert posture | Individual changes posture, raising the front of the body toward a disturbance. State behavior. Measured in seconds. |
| Self-grooming | Self-grooming | Individual searches own fur using one or both hands or mouth. State behavior. Measured in seconds. |
| Scratching | Scratching | Repeatedly rubbing an area with either hand or foot or rubbing against a tree. State behavior. Measured in seconds. |
| Vigilance | General scanning behavior | Individual looks around, sometimes pausing another activity. State behavior. Measured in seconds. |
|  | Playback / researcher directed vigilance | The individual looks toward the speaker/researcher. State behavior. Measured in seconds. |
|  | Head Turn | Individual changes the direction of gaze (moves head position). Point behavior. Number of head turns counted. |
| Researcher directed aggression | Playback / Researcher directed aggression | Individual directs aggressive behavior to the researcher/speaker. Displays genitals (if close may excrete anal fluid). State behavior. Measured in seconds. |
| Out of View | Obscured | The individual is fully or partly obscured, so all behaviors may not be determined. State behavior. Measured in seconds. |
|  | Unknown location | Camera has been unable to follow marmoset so location is temporarily unknown. State behavior. Measured in seconds. |
|  | Absent | Individual has left the area or cannot be located for >20 seconds during the trial and does not return before the end of the trial. Binomial (absent / not absent). Calculated after all other behaviors categorized. |
| Technical error | Technical error | The behavior is unable to be categorized due to an error with the video e.g., unfocused or moving. State behavior. Measured in seconds. |
